# Management of Acute Colchicine Poisoning With Multiple Organ Dysfunction Syndrome: A Case Report

**DOI:** 10.1002/ccr3.73473

**Published:** 2026-09-07

**Authors:** Jintao Luo, Suying Ding, Lei Xu, Sheng Pu, Youxue Bao, Conglin Qin, Fenshuang Zheng

**Affiliations:** ^1^ Kunming Medical University Kunming China; ^2^ The Affiliated Hospital of Yunnan University Kunming China; ^3^ Dongchuan District People's Hospital Kunming China; ^4^ Luxi County People's Hospital Yunnan China

**Keywords:** acute poisoning, blood purification, bone marrow suppression, colchicine, multiple organ dysfunction

## Abstract

Colchicine is a microtubule‐disrupting alkaloid used in the treatment of gout and familial Mediterranean fever. It has a narrow therapeutic window, and overdose is associated with a high risk of fatal toxicity. Colchicine poisoning may initially present with gastrointestinal symptoms resembling acute gastroenteritis, which can make early recognition challenging. The clinical course may deteriorate rapidly, with severe cases involving multiple organ dysfunction. As no specific antidote is currently available, treatment primarily consists of supportive and symptomatic management. Here, we report the clinical course and outcome of a young woman with acute colchicine poisoning. This case highlights the importance of early recognition, close monitoring of organ function, and timely supportive management in patients with severe colchicine poisoning.

## Key Clinical Message

Colchicine may induce life‐threatening multisystem organ dysfunction at doses marginally below the conventionally cited threshold of 0.5 mg/kg. In the absence of a specific antidote, early recognition and serial organ‐function monitoring are critical, while the clinical benefit of delayed gastrointestinal decontamination and extracorporeal therapies in altering the natural course of toxicity remains undefined.

## 1 Introduction

Colchicine is a natural tricyclic alkaloid originally extracted from plants such as *Colchicum autumnale* . Since its first isolation and naming by French chemists Pierre Joseph Pelletier and Joseph Bienaimé Caventou in 1820 [1], it has been used in medicine for over a century. Modern clinical applications primarily include the treatment of acute gout, familial Mediterranean fever, and certain inflammatory diseases [2]. The drug is characterized by a narrow therapeutic window and high toxicity. While relatively safe at therapeutic doses, overdose can cause severe systemic toxic reactions. Although colchicine poisoning is rare, it progresses rapidly, evolving from gastrointestinal symptoms to bone marrow suppression, myocardial injury, and Multiple Organ Dysfunction Syndrome (MODS) within a short period, potentially leading to death. Currently, there is no specific antidote for colchicine poisoning, and management primarily relies on early recognition, close monitoring, and supportive care [3]. Here, we report a case of acute colchicine poisoning with multiorgan dysfunction following a large oral ingestion, describing the patient's clinical manifestations, treatment course, and outcome.

## 2 Case Presentation

### 2.1 Case History and Examination

A 19‐year‐old female weighing 45 kg, with a previously healthy medical history, presented with acute poisoning after ingesting approximately 40 tablets of colchicine (0.5 mg/tablet, total dose 20 mg, approximately 0.44 mg/kg). Approximately 6 h post‐ingestion, the patient developed severe abdominal pain, nausea, and vomiting. She presented to a local hospital where examinations indicated acute liver injury and coagulopathy. The initial hospital performed gastric lavage and provided supportive management, driven by the possibility of a large ingested dose and residual colchicine in the gastrointestinal tract. As the patient's condition progressively deteriorated, she was transferred to our tertiary care center for further evaluation and advanced supportive management.

Upon arrival at the emergency department, the patient reported relief from nausea and vomiting, but persistent abdominal pain remained. Physical examination revealed: T 36.6°C, R 18 bpm, P 78 bpm, BP 124/83 mmHg, SpO_2_ 95%. Abdominal examination showed mild tenderness in the epigastric and periumbilical regions.

### 2.2 Laboratory Findings and Diagnosis

**FIGURE 1:**
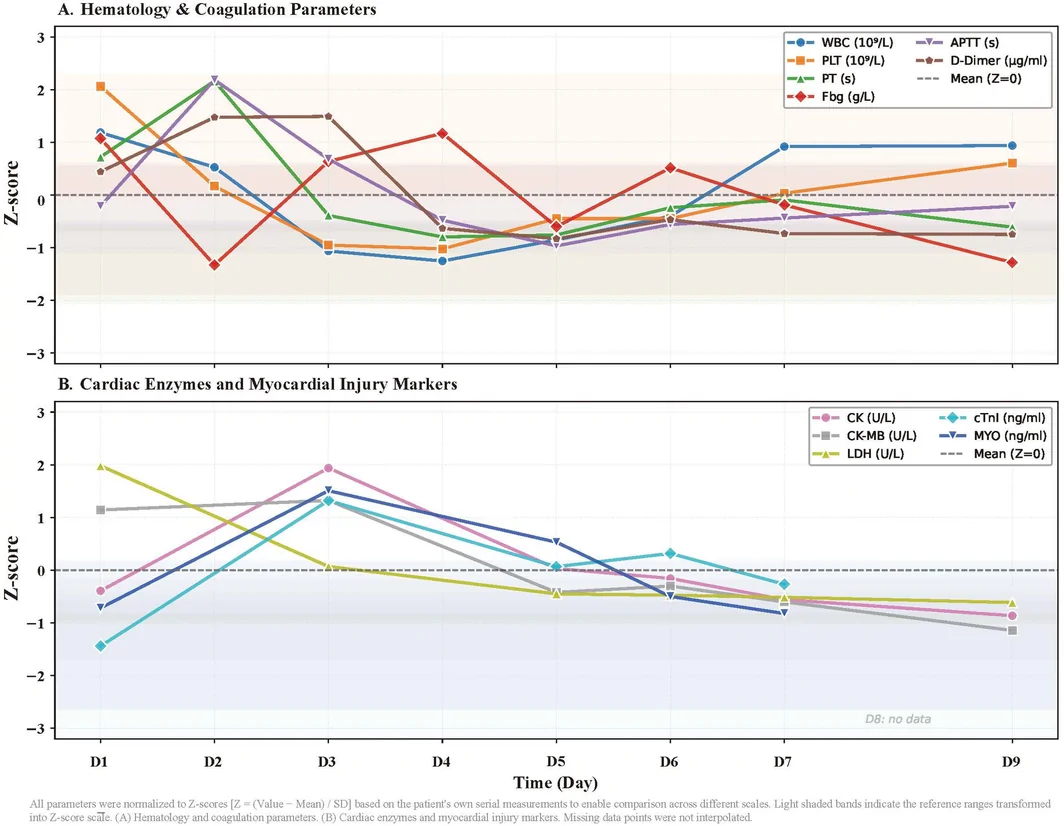
Dynamic changes in laboratory parameters during hospitalization.

Laboratory studies obtained on admission revealed: AST 204 U/L, ALT 28 U/L, CK 299 U/L, CK‐MB 47 U/L, LDH 2586 U/L, cTnI 0.014 ng/mL, and myoglobin 95.7 ng/mL. Arterial blood gas analysis was consistent with metabolic acidosis. These results, considered alongside the clinical history, led to a provisional diagnosis of acute colchicine poisoning with hepatic injury. Serial laboratory findings during hospitalization and the temporal trends of selected parameters are detailed in Table 1 and Figure 1, respectively.

**TABLE 1:** Selected laboratory findings during hospitalization.

Time	D1	D2	D3	D4	D5	D6	D7	D9	Reference range
WBC (10^9^/L)	26.44	19.83	3.89	2.00	5.99	10.53	23.77	23.96	3.5–9.5
HB (g/L)	164	133	94	86	65	97	103	101	115–150
PLT (10^9^/L)	328	143	34	27	83	83	130	186	125–350
AST (U/L)	204	179	212	—	57	59	33	25	13–35
ALT (U/L)	28	24	27	—	35	54	49	37	7–40
Cr(μmol/L)	59	43	44	—	33	35	33	37	41–73
CK (U/L)	299	—	1388	—	497	409	221	78	40–200
CK‐MB (U/L)	47	—	50	—	21	23	18	9	0–25
LDH (U/L)	2586	—	1000	—	569	548	515	433	120–250
CTNI (ng/ml)	0.014	—	0.047	—	0.032	0.035	0.028	—	< 0.026
MYO (ng/ml)	95.7	—	207.4	—	158.4	106.7	90.5	—	0–140.1
PT(s)	16.5	20.4	13.5	12.4	12.5	13.9	14.3	12.9	9.0–13.0
INR	1.46	1.82	1.18	1.08	1.09	1.22	1.25	1.13	0.8–1.2
Fbg (g/L)	4.44	2.46	4.08	4.52	3.06	3.98	3.4	2.5	2.0–4.0
TT (s)	18.7	45.5	23.8	16.9	17.4	17.1	17.9	17.8	14.0–21.0
APTT (s)	27.3	39.1	31.7	26	23.6	25.6	26.2	27.3	25.0–31.3
DD(μg/ml)	18.1	30.6	30.8	5.1	2.7	7.1	3.9	3.7	< 1.0

### 2.3 Initial Management

To minimize further gastrointestinal absorption and enhance fecal elimination of the toxin, single‐dose activated charcoal (SDAC) was promptly administered orally, with concurrent cathartic therapy. Details of the major pharmacological interventions administered during hospitalization are summarized in Table 2. Concurrently, blood purification therapy (CVVH+HP mode, HA330 perfusion device, blood flow 150 mL/min, anticoagulation with trisodium citrate) and plasma exchange (total replacement volume: 1500 mL of fresh frozen plasma [FFP], blood group O, RhD‐positive; anticoagulation with unfractionated heparin) were performed. Considering the multi‐organ damage, symptomatic supportive care and organ protection measures were implemented, including creatine phosphate to improve myocardial energy metabolism and polyene phosphatidylcholine combined with acetylcysteine for hepatoprotection.

**TABLE 2:** Summary of major pharmacological interventions during hospitalization.

Medication	Dose	Route	Frequency	Duration
Sodium phosphate powder combined	21.6:43 g/dose	Oral administration	q12h	4 days
Activated charcoal	25 g/dose	Oral administration	q12h	4 days
Creatine phosphate	1 g/day	Intravenous infusion	qd	9 days
Polyene phosphatidylcholine	930 mg/dose	Intravenous infusion	bid	7 days
Acetylcysteine	8 g/day	Intravenous infusion	qd	9 days
Recombinant human thrombopoietin	15,000 U/day	Subcutaneous injection	qd	4 days
Human granulocyte colony‐stimulating factor	200 μg/day	Subcutaneous injection	qd	4 days
Methylprednisolone sodium succinate	500 mg/day	Intravenous infusion	qd	3 days
Human immunoglobulin	20 g/day	Intravenous infusion	qd	4 days

### 2.4 Multisystem Supportive Care

**FIGURE 2:**
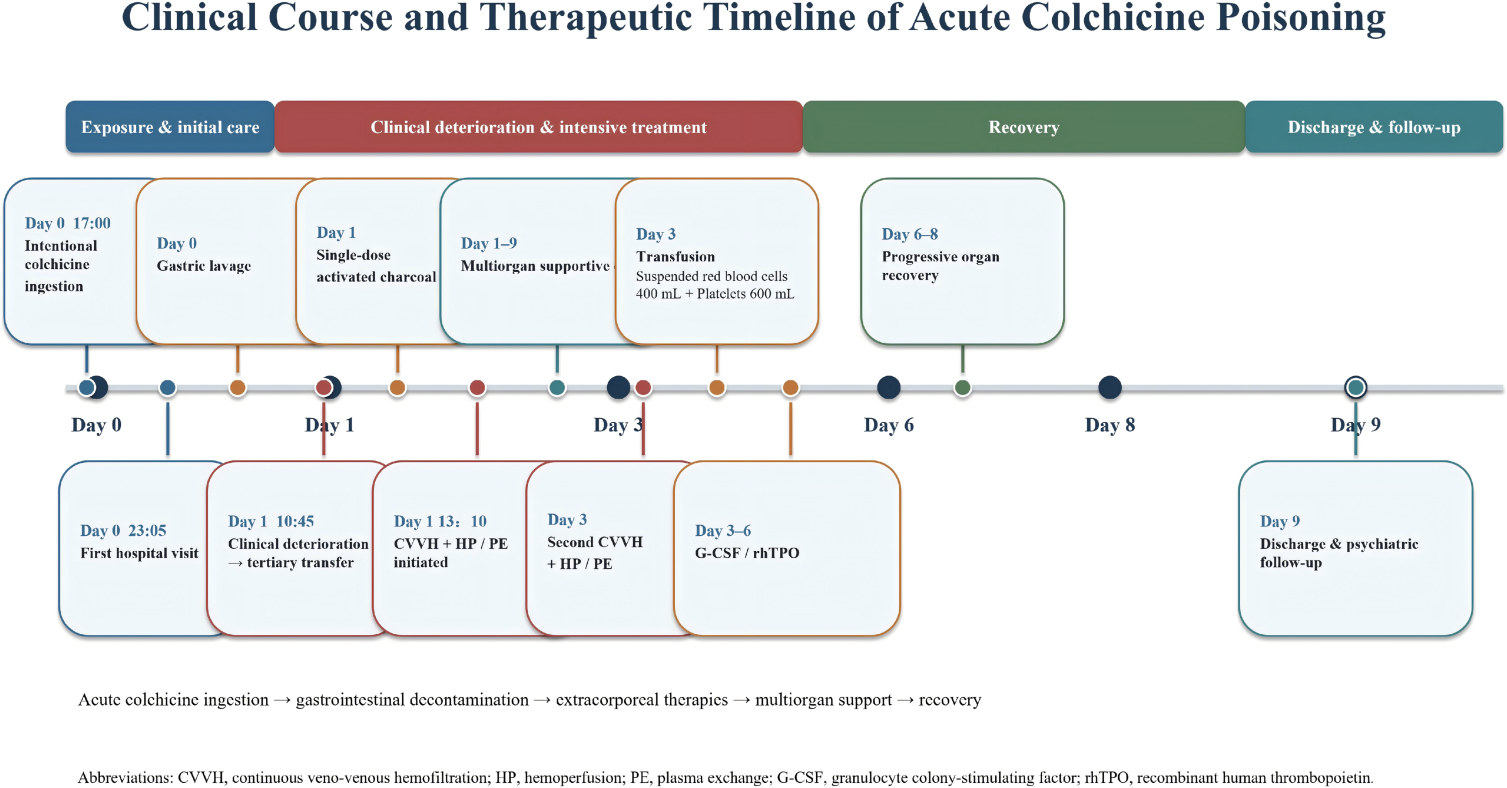
Clinical course and therapeutic timeline of acute colchicine poisoning.

On hospital day 3, the patient developed palpitations, dyspnea, muscle weakness, myalgia, active epistaxis, and gastrointestinal bleeding. Cardiac monitoring showed sinus tachycardia. Repeat laboratory testing showed decreases in WBC count, PLT count, and hemoglobin (HB) to 3.89 × 10^9^/L, 34 × 10^9^/L, and 94 g/L, respectively. Meanwhile, CTNI, CK, and D‐dimer increased to 0.047 ng/mL, 1388 U/L, and 30.8 μg/mL, respectively, while fibrinogen (Fbg) was 4.08 g/L. These findings were accompanied by progressive cytopenias, elevated myocardial injury markers, and coagulation abnormalities. Despite the marked elevation in D‐dimer, the patient did not meet the diagnostic criteria for overt disseminated intravascular coagulation (DIC) based on the Fbg level and International Society on Thrombosis and Hemostasis (ISTH) score. Multisystem abnormalities subsequently developed during the clinical course. Immediate interventions included recombinant human thrombopoietin and human granulocyte colony‐stimulating factor to promote hematopoietic recovery, and methylprednisolone sodium succinate and human immunoglobulin for immune modulation. To address active bleeding, 600 mL of platelets and 400 mL of suspended red blood cells were transfused to correct cytopenia and improve coagulation. For worsening dyspnea and myocardial injury, oxygen therapy via face mask (3–5 L/min) was administered to improve oxygenation, with continued adjunctive phosphocreatine sodium to support myocardial metabolism. Furthermore, to accelerate toxin clearance, blood purification and plasma exchange were repeated using the same parameters and mode. By Day 6, the patient's hemogram gradually recovered, and liver function, myocardial enzyme profile, and CTNI levels declined, indicating gradual restoration of multi‐organ function. Following G‐CSF administration from hospital Days 3 to 6, leukocytosis was observed on Days 7 to 9. No clinical or microbiological evidence of infection was identified during this period. The timeline of the patient's clinical course and management is detailed in Figure 2.

### 2.5 Outcome and Follow‐Up

On Day 9 of treatment, the patient's symptoms significantly improved and met discharge criteria. At discharge, the patient underwent a psychiatric evaluation including suicide risk assessment, received supportive counseling, and was advised to undergo regular follow‐up of liver and renal function, coagulation parameters, and outpatient mental health care. Following discharge, telephone follow‐ups were conducted monthly for 3 months by our department. Psychiatric follow‐up spanned 6 months, with weekly outpatient visits during months 1–2 and monthly visits during months 3–6. No neuropsychiatric sequelae, hematological abnormalities, or chronic hepatic or renal dysfunction were observed during follow‐up, and the patient's prognosis was favorable.

## 3 Discussion

After a large single ingestion of colchicine, the patient initially presented with gastrointestinal symptoms, followed by bone marrow suppression and MODS. The patient's condition gradually improved during intensive supportive and comprehensive treatment, and she ultimately recovered. The clinical course in this case highlights the potential for severe colchicine poisoning to evolve from early gastrointestinal manifestations to multisystem involvement, emphasizing the need for close monitoring and timely supportive management.

Colchicine has a narrow therapeutic window, and the risk of toxicity increases with increasing exposure. In this case, the estimated dose was 20 mg, equivalent to approximately 0.44 mg/kg. An intake of ≥ 0.5 mg/kg can trigger severe systemic poisoning, with mortality risk increasing significantly at doses > 0.8 mg/kg [4]. However, prior case reports suggest that the dose–severe toxicity may occur even when the ingested dose falls below the conventionally defined high‐risk threshold. Parekh et al. reported a case of severe poisoning in a 31‐year‐old female after ingesting 6–12 mg (approx. 0.09–0.17 mg/kg) [5], while Mingjie Fu et al. reported a critical case in a patient with chronic kidney disease after ingesting 0.17 mg/kg [6]. Although the dose in this case was below the traditionally defined high‐risk range, the patient still exhibited the classic triphasic course of poisoning and progressed to life‐threatening MODS. This indicates that assessing severity based solely on ingested dose has significant limitations. Based on this case and previous studies, we suggest that high vigilance is warranted for any suspected colchicine poisoning to avoid treatment delays due to underestimation of the dose.

Colchicine binds to tubulin and inhibits microtubule polymerization, thereby interfering with mitotic spindle formation and cell division [3, 4]. Tissues with high proliferative activity, such as the gastrointestinal tract and bone marrow, are particularly vulnerable to this effect, contributing to the multisystem toxicity observed in severe colchicine poisoning. Colchicine exerts its toxic effects primarily through disruption of microtubule function. By binding to tubulin, it inhibits microtubule polymerization, resulting in impairment of cytoskeletal integrity and cellular function. In skeletal muscle cells and cardiomyocytes, this disruption can lead to direct cytotoxic injury. In addition, colchicine disrupts cytoskeletal dynamics in neutrophils, thereby impairing chemotaxis, adhesion, and phagocytic function [3, 7, 8, 9, 10].

Colchicine poisoning typically follows a classic triphasic clinical course: Phase I (0–24 h) is dominated by gastrointestinal symptoms such as nausea, vomiting, and abdominal pain; Phase II (1–7 days) manifests as multi‐organ dysfunction including bone marrow suppression and myocardial injury; Phase III (> 7 days) is characterized by hematologic recovery and gradual improvement in organ function. On Day 3, the patient developed bone marrow suppression with hemorrhagic manifestations, accompanied by concurrent evidence of skeletal muscle, hepatic, and cardiovascular injury. Notably, renal function remained preserved throughout the observed course. Concurrently, poisoning‐related liver injury causes coagulopathy, and gastrointestinal mucosal injury further increases bleeding risk [11]. In severe cases, systemic inflammatory response and tissue damage may induce DIC, contributing to complex hemorrhagic presentations [12, 13].

The leukocytosis observed on hospital Days 7 to 9 following G‐CSF administration may, at least in part, have reflected the pharmacological effect of G‐CSF; however, this association cannot be definitively established. Myocardial injury is one of the major complications of colchicine poisoning. On admission, the patient exhibited elevated CK and LDH levels, whereas CTNI and MYO remained within the normal range. Serial monitoring demonstrated that CTNI became elevated for the first time on hospital Day 3, suggesting that myocardial involvement developed during disease progression. This phenomenon may be related to colchicine‐induced disruption of microtubule dynamics, which subsequently impairs the electrophysiological stability and contractile function of cardiomyocytes [3]. Although this patient only exhibited sinus tachycardia, without timely intervention, it could have progressed to severe arrhythmias or acute heart failure. Therefore, patients with abnormal myocardial enzymes require intensive ECG monitoring and aggressive supportive care.

Following oral administration, colchicine is rapidly absorbed from the jejunum and small intestine, reaching peak plasma concentrations within 0.5–3 h. It is primarily excreted via the biliary route, with approximately 10%–20% eliminated unchanged by the kidneys [3]. Because colchicine undergoes enterohepatic circulation, drug excreted into the intestine via the bile can be reabsorbed into the systemic circulation [3], resulting in a secondary peak in plasma colchicine concentrations. Gastric lavage was performed at the initial hospital. Its potential benefit in removing residual drug from the stomach is generally considered to be greatest when performed within 1–2 h after ingestion, whereas evidence supporting its use at later time points is limited [4]. After transfer to our hospital, the patient received SDAC, together with CVVH, HP, and PE. In vitro studies have suggested that activated charcoal has a relatively high affinity for colchicine and may theoretically reduce intestinal reabsorption by interfering with enterohepatic circulation [14]. However, the available evidence has primarily supported its use within 4 h of ingestion [15], and the patient presented well beyond this time window. Therefore, the specific clinical benefit of delayed single‐dose activated charcoal in this case remains uncertain.

Colchicine has a molecular weight of approximately 399.44 Da, a relatively large apparent volume of distribution (7–21 L/kg), and extensive tissue binding, which may limit the efficiency of its removal by conventional hemodialysis or a single extracorporeal blood purification session [14, 16, 17]. Previous observational studies have suggested that early combined CVVH and PE may be associated with longer survival; however, the causal benefit of this approach remains uncertain and requires further evidence [18]. One study reported a possible secondary peak in plasma colchicine concentrations approximately 4 h after a single session of plasma exchange, suggesting that the need for repeated extracorporeal blood purification should be assessed cautiously according to the clinical course and pharmacokinetic characteristics of the drug [19]. In the absence of a specific antidote for colchicine poisoning, the mainstay of treatment remains comprehensive supportive care. The current role of extracorporeal blood purification is primarily organ support rather than established removal of colchicine from the body [20, 21]. PE, hemodialysis (HD), continuous renal replacement therapy (CRRT), and other extracorporeal life‐support techniques have been reported in individual cases of severe colchicine poisoning; however, their effects on colchicine removal and clinical outcomes have not been established by high‐quality clinical evidence [17, 18, 19, 22]. The patient underwent a second session of CVVH combined with PE on hospital Day 3, with concurrent supportive management of organ dysfunction. Given the relatively large apparent volume of distribution and substantial tissue accumulation of colchicine, the extent to which extracorporeal blood purification contributed to colchicine removal cannot be precisely quantified. The favorable outcome in this patient should therefore be interpreted as the result of early comprehensive supportive care together with recovery of organ function, rather than being attributed solely to extracorporeal blood purification.

In summary, early recognition and appropriate supportive care remain central to the management of acute poisoning. Gastric lavage is generally considered only within the first hour after ingestion in selected patients, whereas SDAC is typically considered within 4 h, depending on the clinical circumstances. The benefit of delayed gastrointestinal decontamination remains uncertain and is not well supported by current evidence. PE, HD, CRRT, and extracorporeal membrane oxygenation (ECMO) may be considered in selected patients with severe complications or organ dysfunction; however, evidence regarding their efficacy for toxin removal or improvement in clinical outcomes remains limited. Management should therefore be individualized according to the clinical course, severity of poisoning, and organ dysfunction, with multidisciplinary assessment when advanced supportive therapies are being considered.

## 4 Limitations

This case has several limitations. First, colchicine concentrations in blood or urine were not measured because quantitative testing was unavailable at our institution. Consequently, the contribution of PE and CVVH to colchicine elimination could not be directly demonstrated. Second, as this is a retrospective single‐case report, the effectiveness of individual therapeutic interventions and their contribution to the patient's recovery cannot be established.

## 5 Conclusion

The early manifestations of colchicine poisoning are nonspecific and may mimic acute gastroenteritis, frequently contributing to diagnostic delay. Toxicity can progress rapidly, with multiorgan failure documented even at doses below the conventionally accepted lethal threshold. In the absence of a specific antidote, this case highlights that early recognition, serial organ‐function monitoring, and timely intensive supportive care remain central to the management of such patients.

## Author Contributions

**Jintao Luo:** writing – original draft, data curation. **Suying Ding:** writing – original draft, data curation. **Lei Xu:** writing – review and editing. **Sheng Pu:** writing – review and editing. **Youxue Bao:** writing – review and editing. **Conglin Qin:** writing – review and editing. **Fenshuang Zheng:** writing – review and editing, supervision.

## Funding

This work was supported by Key Laboratory of Emergency Medicine of Yunnan Province, 202449CE340015. Expert Workstation of Yu Xuezhong, Yunnan Province, 202505AF350060.

## Ethics Statement

As a single‐case report with the patient's signed consent, no other ethical review was required.

## Consent

Informed written consent was obtained from the patient to publish this case report. A copy of the written consent form is available for review by the journal editor.

## Conflicts of Interest

The authors declare no conflicts of interest.

## Data Availability

The data that support the findings of this study are available on request from the corresponding author. The data are not publicly available due to privacy or ethical restrictions.
